# Endoscopic Treatment of T1 Colorectal Cancer

**DOI:** 10.3390/cancers15153875

**Published:** 2023-07-30

**Authors:** Klaus Metter, Stephanie Ellen Weißinger, Alinda Várnai-Händel, Karl-Ernst Grund, Franz Ludwig Dumoulin

**Affiliations:** 1 Klinik für Gastroenterologie, Hepatologie und Diabetologie, Alb Fils Kliniken, Klinik am Eichert, Eichertstraße 3, D-73035 Göppingen, Germany; 2 Institut für Pathologie, Alb Fils Kliniken, Klinik am Eichert, Eichertstraße 3, D-73035 Göppingen, Germany; stephanie.weissinger@af-k.de; 3 Institut für Pathologie Bonn Duisdorf, Heilsbachstr. 11, D-53125 Bonn, Germany; varnai-haendel@patho-bonn.de; 4 Experimentelle Chirurgische Endoskopie (CETEX), Universitätsklinikum Tübingen, Waldhörnlestraße 22, D-72072 Tübingen, Germany; k.e.grund@web.de; 5 Innere Medizin/Gastroenterologie, Gemeinschaftskrankenhaus Bonn, Prinz Albert Str. 40, D-53113 Bonn, Germany; f.dumoulin@gk-bonn.de

**Keywords:** colorectal cancer, T1 cancer, endoscopic resection, endoscopic mucosal resection, EMR, endoscopic submucosal dissection, ESD, endoscopic submucosal resection, ESR, endoscopic full thickness resection, EFTR, lymph-node metastasis risk

## Abstract

**Simple Summary:**

Endoscopic resection of early-stage colorectal cancer is sufficient if histopathology indicates a low risk for lymph-node metastasis. Among the factors associated with lymph-node metastasis, tumor infiltration depth into the submucosal layer seems to carry the lowest risk, particularly in the absence of other risk factors. Unfortunately, with current resection techniques, the quantity and quality of the submucosal layer are often insufficient. Thus, histopathology may become unreliable, and unnecessary surgery may result. A resection strategy using novel devices designed for endoscopic resection of a maximum quantity and quality of the submucosal layer should provide an advantage.

**Abstract:**

Commonly accepted criteria for curative resection of T1 colorectal cancer include R0 resection with horizontal and vertical clear margins (R0), absence of lympho-vascular or vessel infiltration (L0, V0), a low to moderate histological grading (G1/2), low tumor cell budding, and limited (<1000 µm) infiltration into the submucosa. However, submucosal infiltration depth in the absence of other high-risk features has recently been questioned as a high-risk situation for lymph-node metastasis. Consequently, endoscopic resection techniques should focus on the acquisition of qualitatively and quantitively sufficient submucosal tissue. Here, we summarize the current literature on lymph-node metastasis risk after endoscopic resection of T1 colorectal cancer. Moreover, we discuss different endoscopic resection techniques with respect to the quality of the resected specimen.

## 1. Introduction

Colorectal cancer (CRC) is one of the most frequent malignancies in Western societies [1]. It most commonly evolves from benign precursor lesions (adenomas) over a long time period. This window of opportunity calls for preventive measures and various screening strategies ranging from fecal occult blood tests to scheduled endoscopic screening have been implemented. Recently, colonoscopy screening has been evaluated in a prospective randomized study and was shown to reduce mortality by 50% in the per protocol analysis [2]. Screening colonoscopy not only allows identification and removal of precursor lesions (i.e., adenomas) but it also detects an increasing number of early cancers—some of them diagnosed only after endoscopic removal as ‘polyp cancers’ [3]. 

## 2. Endoscopic Evaluation of Colorectal Lesions—Not Always Perfect

Endoscopic resection of T1 CRC is accepted as an oncological sufficient treatment if histopathology confirms a low lymph-node metastasis risk. Current guidelines recommend a thorough evaluation of all colorectal lesions before endoscopic resection to choose the appropriate resection technique in case of suspicious lesions [4]. High-definition endoscopy in combination with (virtual or dye) chromoendoscopy is recommended to evaluate surface and vessel structure and to predict the presence of submucosal infiltration in flat or sessile colorectal lesions prior to endoscopic resection, e.g., using the NICE (narrow band imaging international colorectal endoscopic) or JNET (Japanese NBI expert) classification [5,6]. In larger rectal lesions, additional imaging, particularly endoscopic ultrasound, may be considered [7]. In addition to surface and vessel pattern the size, morphology and localization of the lesion are important. In a large meta-analysis of over 5000 adenomas, the risk for submucosal invasive cancer increased from 9.2% for lesion sizes between 20 and 30 mm to 16.5% for lesions larger than 30 mm [8]. The risk for submucosal invasive cancer also varied greatly with the morphology and was lowest for lateral spreading tumors (LST) of granular homogeneous type and highest for LST nongranular type with pseudo-depression. There also was a higher risk for cancer in distal versus proximal localized lesions. The meticulous evaluation of a colorectal lesion is important to choose the correct resection strategy. It should minimize cases of unnecessary surgery as a consequence of a poor resection technique (resulting in a specimen that is inappropriate for accurate histopathology) and should also prevent potentially harmful attempts to resect lesions endoscopically that are not suitable for local treatment [9]. 

Endoscopic diagnosis, however, even with the use of magnifying endoscopy or the assistance of artificial intelligence systems, is far from perfect [10,11,12,13]. In a systematic review and meta-analysis, the sensitivity/specificity of virtual chromoendoscopy were 85%/94% for the diagnosis of submucosal invasive cancer and 77%/98% for the diagnosis of deep submucosal invasion [14]. Endoscopic optical diagnosis aims at the diagnosis of submucosal invasive cancer and quantifying the depth of submucosal invasion to choose the appropriate resection technique [15]. However, depth of submucosal invasion may not be a good predictor of lymph-node metastasis in the lower gastrointestinal tract [16]. Moreover, in daily routine, some cancers are not suspected at the time of polypectomy but diagnosed only afterward on histopathology. Given this diagnostic uncertainty, an ideal endoscopic resection technique, particularly for rectal lesions >20 mm that have a higher risk for initial submucosal invasive cancer than lesions in the proximal colon, should obtain a one-piece specimen with well-defined lateral margins and the highest possible quality and quantity of submucosal tissue. The specimen resected by such a technique would allow the optimal evaluation of resection margins even in cases of unexpected submucosal invasive cancer and, thus, maximize the chance of a curative endoscopic resection. In addition, such a resection technique should come with a low rate of complications, particularly a low perforation rate, to avoid potential tumor cell spread [7,17]. 

## 3. T1 CRC—Not All Criteria for ‘Curative’ vs. ‘Noncurative’ Resection Are Equally Important

In the case of endoscopic resection of T1 CRC, the final diagnosis of ‘curative’ (i.e., low lymph-node metastasis risk) versus ‘noncurative’ resection will depend on histological analysis of the resected specimen. Commonly accepted criteria for ‘curative’ endoscopic resection include (i) a complete resection (ideally as one-piece/en bloc resection) with horizontal and vertical margins free of tumor infiltration (R0), (ii) the absence of lymphatic or blood vessel infiltration (L0, V0), (iii) a low to moderate histological grading (G1/2), and (iv) a limited (<1000 µm) tumor infiltration depth into the submucosa [7,18,19,20]. In addition, high-grade tumor budding (defined as single cells or cell clusters at the tumor invasion front) has been established as an important independent predictor of lymph-node metastasis [21,22]. Other factors such as tumor localization in the sigmoid/rectum were found to be associated with a higher lymph-node metastasis risk [23,24,25] but conflicting results have also been reported [26]. The concept of endoscopic resection of T1CRC was confirmed in a recent meta-analysis of 71 studies including more than 5000 patients. The pooled cumulative incidence of recurrence was 3.3% (local recurrence 1.9%; metastatic disease 1.6%) with different recurrence rates for low- versus high-risk lesions (0.7% versus 7%). CRC-related mortality was high (41%) in patients with recurrence [27]. 

Not all of the abovementioned factors defining a ‘noncurative’ resection are equally important. In most studies, lympho-vascular invasion (L1), poor grading, and a high tumor cell budding were strongly associated with lymph-node metastasis. In contrast, submucosal infiltration depth > 1000 µm in the absence of all other high-risk features is probably associated with a relatively low risk of lymph-node metastasis. This was shown in a recent meta-analysis on 67 studies with 21.238 T1CRC patients with an overall lymph-node metastasis rate of 11%. When weighting the different predictive factors, only poor differentiation, a high tumor budding count, and lymph vessel infiltration were significant predictors of lymph-node metastasis. In eight of the included studies comprising a total of 1146 patients, submucosal invasion depth > 1000 µm was the only risk factor, and, in this constellation, the lymph-node metastasis rate was only 2.6% [16]. This finding is supported by more recent studies not included in the meta-analysis (Table 1). 

## 4. Optimizing the Prediction of Lymph-Node Metastasis Risk of T1 CRC

As pointed out, only 11–15% of the patients who undergo surgical resections after an endoscopic ‘noncurative resection’ will actually be diagnosed with lymph-node metastasis [16,28,33]. Different approaches have been undertaken to meet the need for a more accurate prediction of the lymph-node metastasis risk after ‘noncurative’ endoscopic resections (Table 2). Kajiwara et al. analyzed 4673 T1 CRC patients (352 with lymph-node metastasis) and elaborated a nomogram from a multivariate model of a development cohort (*n* = 3080/4673) to predict lymph-node metastasis. Six independent risk factors were identified: female gender, distal tumor location, high tumor grading, lymph vessel infiltration, high tumor cell budding, and submucosal invasion depth > 1000 µm. The nomogram was subsequently used to calculate the lymph-node metastasis risk in the validation cohort (the remaining 1593 patients) and performed far better than the current Japanese and US guidelines [34]. Another large study used data from 3134 patients (10.2% with lymph-node metastasis) as an input for a machine learning system to predict lymph-node metastasis risk [35]. A neuronal network was trained with available clinical data (age, sex, tumor size, location, morphology, lymphatic and vascular invasion, and histologic grade) and validated on a different set of 939 patients. The area under the curve (AUC) for the prediction of lymph-node metastasis of the artificial intelligence system was significantly better than that of current guidelines (AUC 0.83 versus 0.73, *p* < 0.01). This finding could also be reproduced if the analysis was restricted to 517 cases with initial endoscopic resection (AUC 0.84 versus 0.77, *p* = 0.05). Lastly, tissue-based transcriptomic biomarkers and different messenger RNAs were used to predict lymph-node metastasis risk [36]. This study analyzed 330 specimens from patients with high-risk T1 CRC (29 with lymph-node metastasis). A transcriptomic panel consisting of four different micro RNAs and five messenger RNAs was applied to 46 serum samples for training and to an additional 142 serum and matching tissue samples for validation. A risk-stratification model which combined the results of the transcriptomic panel and clinical risk factors identified patients with lymph-node metastasis with high precision and limited potential surgical overtreatment to only 18% of all patients included in the study. By and large, these studies confirm the notion that submucosal infiltration depth per se is a weak risk factor for lymph-node metastasis. 

## 5. Endoscopic Resection Techniques and Specimen Quality

**Figure 1:**
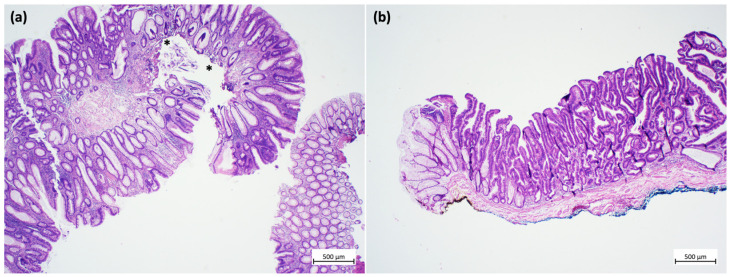
Examples of specimens from endoscopic resections. (**a**) Low-quality specimen: fragmented portions of a tubulo-villous adenoma with low-grade dysplasia. Due to the fragmentation, inking of the resection margins is not possible, the resection status cannot be assessed, and the submucosa cannot be measured. In addition, thermal artefacts are seen in the depth (asterisks) (hematoxylin and eosin stain). (**b**) High-quality specimen: one-piece resection of a tubulo-villous adenoma with serrated morphology and focal high-grade dysplasia. Inking can be performed both for the lateral (yellow) and the vertical (blue) resection margins. Thus, the resection status can be assessed, and the submucosal layer can be measured. Thermal artefacts are not seen. (hematoxylin and eosin stain). Images obtained using an Olympus BX41 microscope, Zeiss digital camera Axiocam105 color and a 2 × 0.06 Plan Neofluar objective (Zeiss Zen core 3.5 software). Image processing was performed with Microsoft PowerPoint, Version 2020.

The quality of a resected specimen is the key to correct histopathology. In particular, a complete resection submucosal layer is desired in cases of submucosal invasion, which, as pointed out above, is probably not an independent risk factor for lymph-node metastasis. The consequences of the quality of a specimen (piecemeal versus one-piece) for subsequent histopathology are shown in Figure 1. In this example, fragmentation and thermic artefacts prevent a reliable evaluation of submucosal infiltration depth and vertical resection margin [45]. 

### 5.1. ESD

While en bloc resection of smaller well lifting lesions can be achieved by EMR, current ESGE guidelines recommend ESD for resection of larger (than 2 cm) colorectal, but particularly rectal, lesions with suspicion of limited submucosal invasion [7]. ESD will reliably achieve a one-piece resection and allows for optimal control of the resection margins. In addition, very good results have been published for the resection of superficially invasive T1CRC [46,47]. ESD may, however, have a problem with the vertical resection margin in cases of deep submucosal invasion. Thus, a recent Western retrospective multicenter study with 207 colorectal ESDs reported a positive vertical margin in 57% of the cases, and a positive vertical margin was the most frequent reason for a ‘noncurative’ endoscopic resection [48]. A possible caveat regarding the vertical resection margin also comes from a small pilot study comparing colorectal specimens obtained by EMR (*n* = 6 benign lesions) versus ESD (*n* = 6, with one case of submucosal invasive cancer) and quite unexpectedly showed a better preserved and thicker submucosal layer in EMR specimens [49,50]. More reassuring are retrospective data from a multicenter study on 126 ESDs performed for lesions suspicious for focal deep submucosal invasion. In this report, en bloc and R0 rates were 96% and 77%, respectively. Moreover, 26% of the resections were curative, 30% showed submucosal invasion depth > 1000 µm with no other high-risk features, and only 17% were clearly noncurative due to lymph vessel infiltration or high budding [51].

### 5.2. Endoscopic Intermuscular Dissection (EID) 

An attempt to optimize the vertical resection margin in the rectum is a recently described technique of EID. This technique dissects the lesions between the outer longitudinal and the inner circular muscle layer. The specimen will, thus, include the complete submucosal layer and allow an optimal histological assessment in cases of submucosal invasive cancer. In a recent prospective cohort study, 67 patients with suspected deeply invasive rectal cancer were treated. The technical success rate for a median lesion size of 25 mm was 96%. EID allowed a high R0 resection rate even for deeply invasive T1 rectal cancers (36/40 cases; 90%) and avoided rectal surgery in nearly half of the patients [52].

### 5.3. Endoscopic Full Thickness Resection (EFTR)

An EFTR would provide an optimal specimen for evaluation of submucosal infiltration depth and vertical margin. The most commonly used tool is the full thickness resection (FTRD) device. Its use is attractive, since the learning curve is relatively short. Moreover, procedure time and perforation rate for non-lifting lesions might be favorable. However, the FTRD device has a limitation for the size of the resected specimen, and its use is restricted to smaller lesions up to 15–20 mm [53,54]. For 92 non-lifting cancerous lesions, the reported R0 rate from the German FTRD study group was 60.9% when FTRD was used as the primary resection method and 87.5% when the method was applied after incomplete resection of a malignant lesion [55]. Data from the Netherlands on 132 primary resections of T1 CRC showed a curative resection rate of 60.8% after excluding deep submucosal invasion as risk factor [56]. 

### 5.4. Endoscopic Submucosal Resection (ESR)

**Figure 2:**
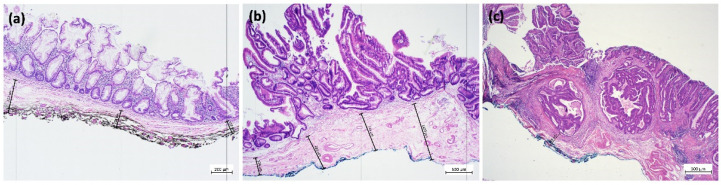
(**a**) EMR en bloc of a sessile serrated lesion with dysplasia with inked deep resection margin (black) (4× magnification, hematoxylin and eosin stain). Grid lines at 1000 µm intervals. Orthogonal to this, submucosa thickness is measured from the lamina muscularis mucosae to the deep vertical resection margin (black). (**b**) ESR en bloc of a tubulo-villous adenoma with serrated morphology and high-grade dysplasia and inked deep resection margin (blue), with measurement as above (hematoxylin and eosin stain). Note the differences in the thickness of the submucosal layer and the quality of the vertical margin in (**a**) (EMR) versus (**b**) (ESR). (**c**) Specimen obtained by ESR for a lesion with invasive carcinoma. En bloc specimen of a tubulo-villous adenoma with serrated morphology and focal high-grade dysplasia with transition into a well-differentiated invasive adenocarcinoma, intestinal type with infiltration of the submucosa. Tumor classification: pT1 (sm1) L0 V0 R0 G1. Inking of the deep resection margin was performed, and the distance of the lesion to the deep resection margin was measured (hematoxylin and eosin stain). Image acquisition and processing as given for Figure 1.

ESR is carried out with specific devices designed to increase quality and quantity of resected submucosal layer with a minimal perforation risk [57,58]. FARIn U is a needle knife attached to an insulated glider for mucosal incision. The flat adenoma resection instrument (FARIn) is a partially insulated snare with a distal ceramic tip for submucosal resection. Similar to hybrid EMR/ESD, ESR consists of a complete mucosal incision followed by submucosal resection, for which the FARIn device is placed around the lesion and firmly pushed *against* the bowel wall during the resection [57,59]. A multicenter study on 93 lesions in various localizations (11 stomach, 25 colon, and 57 rectum) with a median size of 29 mm (range 10–70) showed technical feasibility in all cases. The en bloc and R0 resection rates were 70% and 63%, respectively, with a promising safety profile (two delayed bleedings, one micro-perforation, no need for emergency surgery, and no 30 day mortality); all R1 resections had a positive lateral, not vertical margin [57]. Initial data show more abundant submucosal tissue for ESR than for EMR specimens which is of particular importance in cases of submucosal invasive cancer (Figure 2a–c). A prospective study formally comparing precut EMR and ESR is underway. 

## 6. Conclusions

Endoscopic resection of T1 CRC is accepted as adequate oncological treatment if lymph-node metastasis risk of the resected lesion is low. This is commonly accepted for horizontal and vertical margins free of tumor infiltration, absence of lymphatic or blood vessel infiltration (L0, V0), low to moderate histological grading (G1/2), low tumor cell budding, and a limited (<1000 µm) submucosal tumor infiltration depth. The histological assessment of these features will critically depend on the quality of the resected specimen, which itself depends on the endoscopic resection technique. Since submucosal infiltration depth > 1000 µm in the absence of all other high-risk features is probably associated with a relatively low risk of lymph-node metastasis, a good-quality specimen should include abundant submucosal tissue and minimal thermal artefacts, particularly at the vertical margin. The various resection methods currently available all have their specific benefits and limits; EFTR and EID will provide a full submucosal layer, but EFTR has size limits, and EID is limited to rectal lesions. ESD has no size limits and provides optimal control of lateral margins; it may, however, have limitations with vertical resection margins. ESR will resect close to the proper muscle layer, but it also has a size limit of 40 mm. 

## Figures and Tables

**Table 1:** Recent studies on lymph-node metastasis (LNM) in T1 CRC with deep submucosal invasion in the absence of other high-risk factors.

Study/Year	Population	LNM (per Total Number of Cases)	LNM (sm Infiltration >1000 µm in Isolation)
Yasue et al., 2022 [28]	Endoscopic, surgical	74/864 (8.7%)	1.6%
Rönnow et al., 2022 [29]	Surgical	150/1439 (10%)	7.4%
Yamaoka et al., 2022 [30]	Surgical	70/519 (13.5%)	3.8%
Morini et al., 2022 [31]	Surgical	15/122 (12.5%)	2.9%
Santos-Antunes et al., 2023 [32]	Endoscopic	26/96 (27%)	0.0%

**Table 2:** Selected studies on prediction of lymph-node metastasis risk in T1 CRC.

Study	Method	Number	Result (Prediction LNM Risk)
Yasue et al. [28]	Histology	N = 846	LVI, budding and differentiation: OR 8.09, 1.89, 2.09
Piao et al. [37]	Histology	N = 271	LVI and grading: 0.8% vs. 66.6% LNM
Miyazaki et al. [38]	Exosomal miRNAs from preoperative serum	N = 200	AUC 0.84 for LNM
Kajiwara et al. [34]	Nomogram	N = 4673	SMI, budding, LVI, grading, location, sex
Takamatsu et al. [39]	AI/deep learning from H&E histology	N = 783	AUC 0.76 for LNM
Song et al. [40]	AI/deep learning from H&E histology	N = 400	AUC 0.76 for LNM
Kasahara et al. [41]	AI/deep learning from H&E histology	N = 146	Accuracy 81.8–86.3%
Yan et al. [42]	Nomogram	N = 141	LVI, location, grading AUC 0.89
Wada et al. [36]	Liquid biopsy Micro RNA, mRNA	N = 330	AUC 0.90
Tang et al. [43]	Multivariate analysis	N = 476	Early-onset CRC with higher LNM rate
Kudo et al. [35]	AI/deep learning with various risk factors	N = 3134	AUC 0.83 for cases with initial endoscopic resection (*n* = 517)
Kang et al. [44]	Learning algorithm with various factors	N = 316	AUC 0.76

Abbreviations: AI, artificial intelligence; AUC, area under the curve; CRC, colorectal cancer; H&E hematoxylin/eosin; LNM, lymph-node metastasis; LVI, lymphatic vessel infiltration; SMI, submucosal invasion depth.

## Dedication

This paper is dedicated to the commemoration of Dipl.-Ing. Günter Farin (deceased), the ingenious pioneer of electrosurgery for flexible endocopic therapy.
